# Endothelial cells are an important source of BDNF in rat skeletal muscle

**DOI:** 10.1038/s41598-021-03740-8

**Published:** 2022-01-10

**Authors:** Marina Cefis, Remi Chaney, Aurore Quirié, Clélia Santini, Christine Marie, Philippe Garnier, Anne Prigent-Tessier

**Affiliations:** 1 INSERM UMR1093-CAPS, Université Bourgogne Franche-Comté, UFR Des Sciences de Santé, 21000 Dijon, France; 2 Département Génie Biologique, IUT, 21000 Dijon, France; 3 UFR Des Sciences de Santé, 7 boulevard Jeanne d’Arc, 21078 Dijon, France

**Keywords:** Cell biology, Neuroscience

## Abstract

BDNF (brain-derived neurotrophic factor) is present in skeletal muscle, controlling muscular metabolism, strength and regeneration processes. However, there is no consensus on BDNF cellular source. Furthermore, while endothelial tissue expresses BDNF in large amount, whether endothelial cells inside muscle expressed BDNF has never been explored. The aim of the present study was to provide a comprehensive analysis of BDNF localization in rat skeletal muscle. Cellular localization of BDNF and activated Tropomyosin-related kinase B (TrkB) receptors was studied by immunohistochemical analysis on soleus (SOL) and gastrocnemius (GAS). BDNF and activated TrkB levels were also measured in muscle homogenates using Western blot analysis and/or Elisa tests. The results revealed BDNF immunostaining in all cell types examined with a prominent staining in endothelial cells and a stronger staining in type II than type I muscular fibers. Endothelial cells but not other cells displayed easily detectable activated TrkB receptor expression. Levels of BDNF and activated TrkB receptors were higher in SOL than GAS. In conclusion, endothelial cells are an important and still unexplored source of BDNF present in skeletal muscle. Endothelial BDNF expression likely explains why oxidative muscle exhibits higher BDNF levels than glycolytic muscle despite higher the BDNF expression by type II fibers.

## Introduction

Since its first identification in the brain in 1982^1^, neuronal BDNF (brain-derived neurotrophic factor) was the subject of a plethora of studies that have led to the consensus that neuronal-derived BDNF is crucial for neurogenesis, synaptogenesis, neuroplasticity and cognition^2,3^ through phosphorylation at tyrosine 816 of full-length (FL) tropomyosin-related kinase B (p-TrkB^Tyr816^)^4,5^. However, we know now that BDNF is expressed in other cells than in neurons and therefore plays a more widespread role than initially thought. Skeletal muscle, which is the most abundant tissue in the body, was shown to synthesize BDNF in 1993^6^. The demonstration that physical exercise leads to an elevation of BDNF in active muscle^7^ and the idea that BDNF originating from muscle might be involved in the beneficial effect of EX not only on muscles but also on distant organs explains the regain of interest for muscular BDNF.

Surprisingly, little is known about the cellular localization of BDNF. Available studies focused mainly on muscular fibers, satellite cells and neuromuscular junctions and led to controversial results. For certain authors, BDNF expression is restricted to the former^8,9^, while it is confined to the latter for others^10^. There is also no consensus concerning BDNF localization on the neuromuscular junction^11–13^. Although never explored, BDNF might be also expressed by endothelial cells present in muscle. Indeed, the cerebral and peripheral vasculatures including capillaries contain BDNF with a prominent expression by the endothelium^14–16^. To explore whether endothelial cells present in muscle express BDNF is of great interest as density of capillary network differs between oxidative and glycolytic muscles. In addition, if BDNF is present in endothelial cells, the elevation of circulating BDNF observed during or after exercise might possibly reflect the secretion of BDNF by endothelium and not by that of myofibers as generally thought according to the concept of myokines.

The present study tests the hypothesis that BDNF is expressed by endothelial cells in skeletal muscle and that this expression largely contributes to BDNF levels present within the muscle. For this purpose, we first provide a comprehensive analysis of BDNF localization in skeletal muscle in rats. Then, we compared BDNF content of oxidative muscle soleus (SOL) versus glycolytic muscle gastrocnemius (GAS) since capillary network was denser for the former.

## Results

### Cellular BDNF localization in muscle

**Figure 1:**
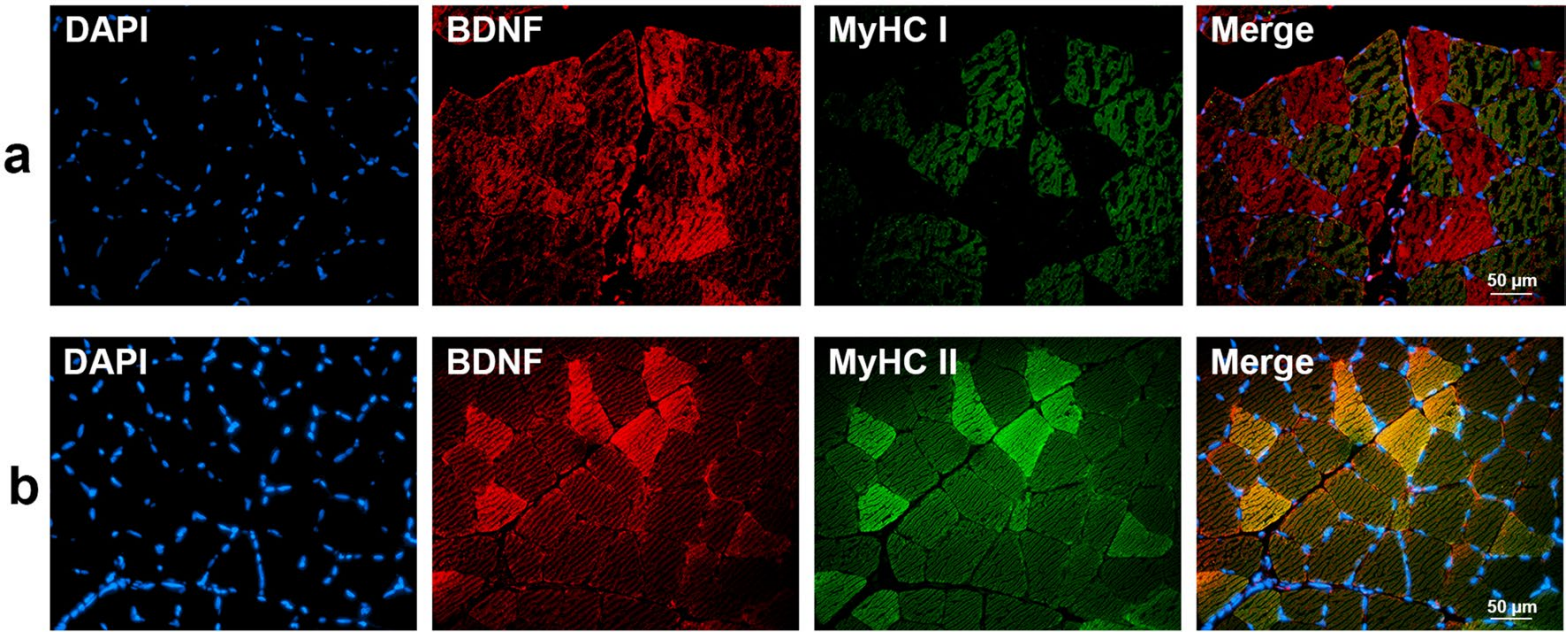
Colocalization of BDNF with slow-type (MyHC I, **a**) and fast-type fibers (MyHC II, **b**) in soleus (SOL) muscle. DAPI (blue) and MyHC I/II (Myosin Heavy chain I/II, green) were used as nuclear and slow I (MyHC I)/fast II (MyHC II) myosin isoform markers, respectively. Red-labelled immunofluorescence represents BDNF. A great BDNF staining in fast-type fibers was visualized in SOL muscle. Similar results were observed in all examinations (*n* = 4). Scale bar 50 µm.

**Figure 2:**
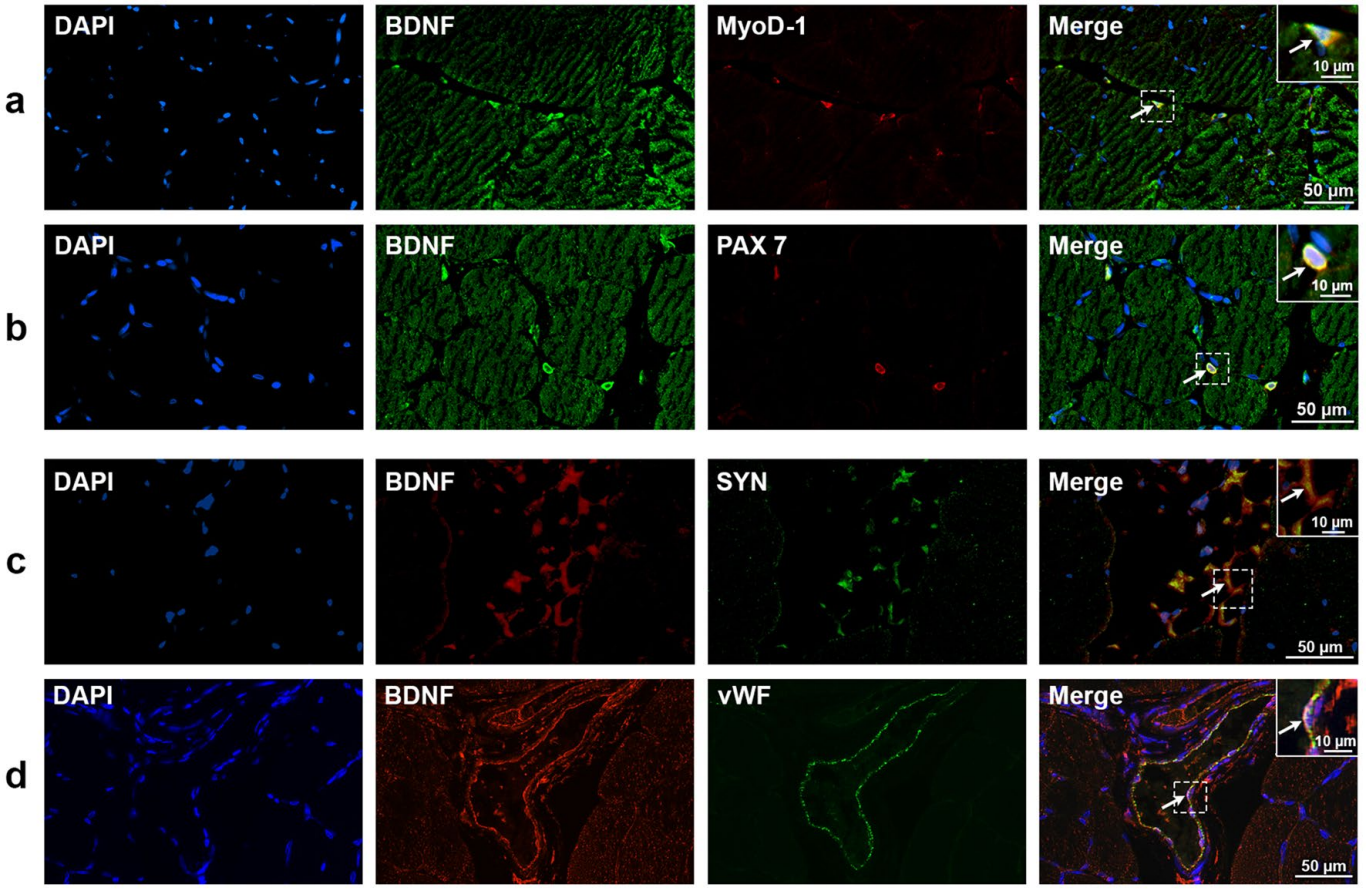
Colocalization of BDNF with specific markers of satellite (MyoD-1, myoblast determination protein 1/PAX 7, Paired box 7) cells, axonal processes of motor neurons (SYN, synaptophysin) and endothelial (vWF, von Willebrand factor) cells in soleus (SOL) muscle. DAPI (blue) was used as nuclear marker. BDNF was stained either in green (**a, b**) or in red (**c, d**), MyoD-1 and PAX 7 were marked in red (**a, b**) and, SYN and vWF in green (**c, d**). Similar results were observed in all examinations (*n* = 4). The inserts show with higher magnification the colocalization of BDNF with each specific marker in presence of DAPI. Scale bar 50 µm (10 µm, for the insert).

Experiments were first conducted on SOL. As shown in Fig. 1, BDNF staining was very weak in type I fibers (Fig. 1a) and by contrast strong in type II fibers (Fig. 1b). BDNF was also present (Fig. 2) in satellite cells as evidenced from a BDNF labelling by cells positive for MyoD-1 (Fig. 2a) and PAX7 (Fig. 2b). Axonal processes of motor neurons also expressed BDNF. Indeed, BDNF colocalized with synaptophysin (Fig. 2c). Finally, cells positive for the specific endothelial marker von Willebrand factor (vWF) exhibited a strong labelling for BDNF (Fig. 2d).

**Figure 3:**
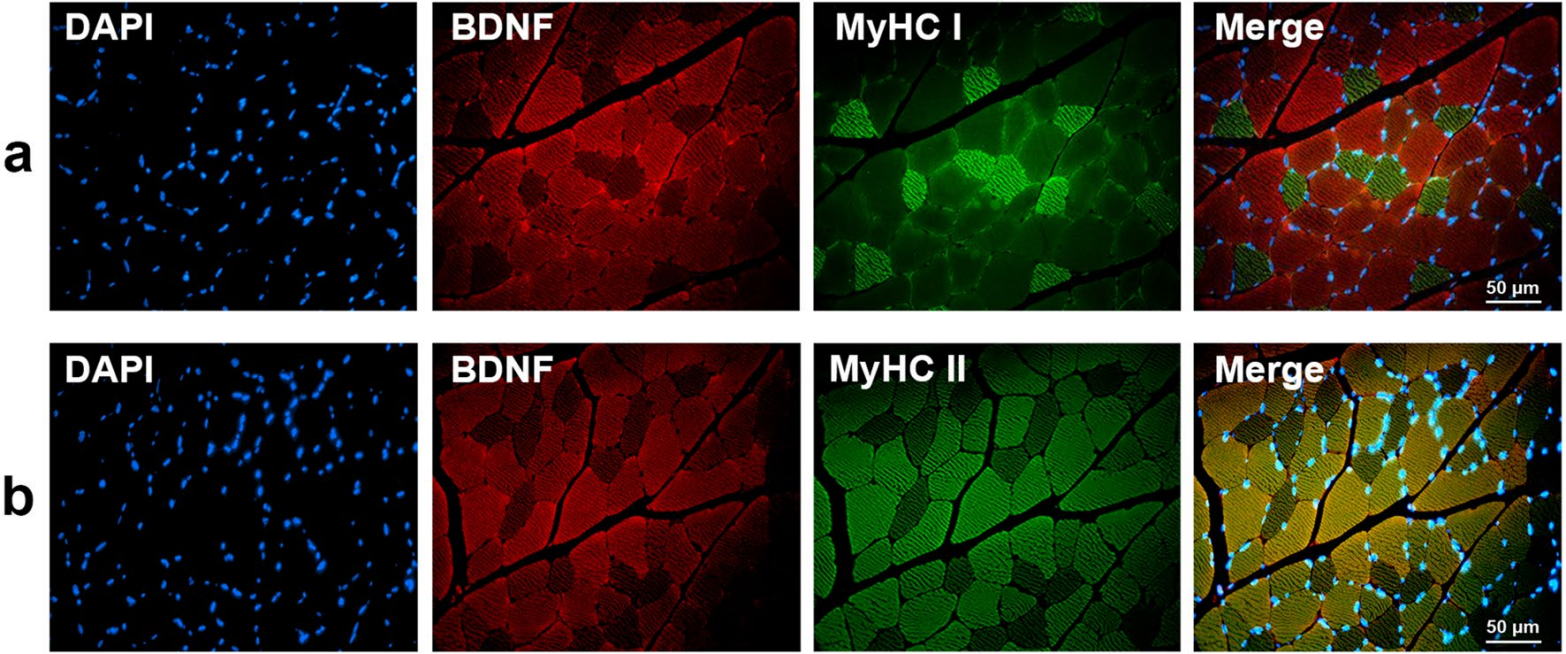
Colocalization of BDNF with slow-type (MyHC I, **a**) and fast-type fibers (MyHC II, **b**) in gastrocnemius (GAS) muscle. DAPI (blue) and MyHC I/II (Myosin Heavy chain I/II, green) were used as nuclear and slow I (MyHC I)/fast II (MyHC II) myosin isoform markers, respectively. Red-labelled immunofluorescence represents BDNF. A great BDNF staining in fast-type fibers was visualized in GAS muscle. Similar results were observed in all examinations (*n* = 4). Scale bar 50 µm.

Experiments were then conducted on GAS. Figure 3 confirmed the high BDNF expression by type II fibers (Fig. 3a) as compared to type I fibers (Fig. 3b). As reported for SOL, BDNF staining was also detected in satellite cells, neurons and endothelial cells (data not shown).

### BDNF levels in SOL versus GAS

**Figure 4:**
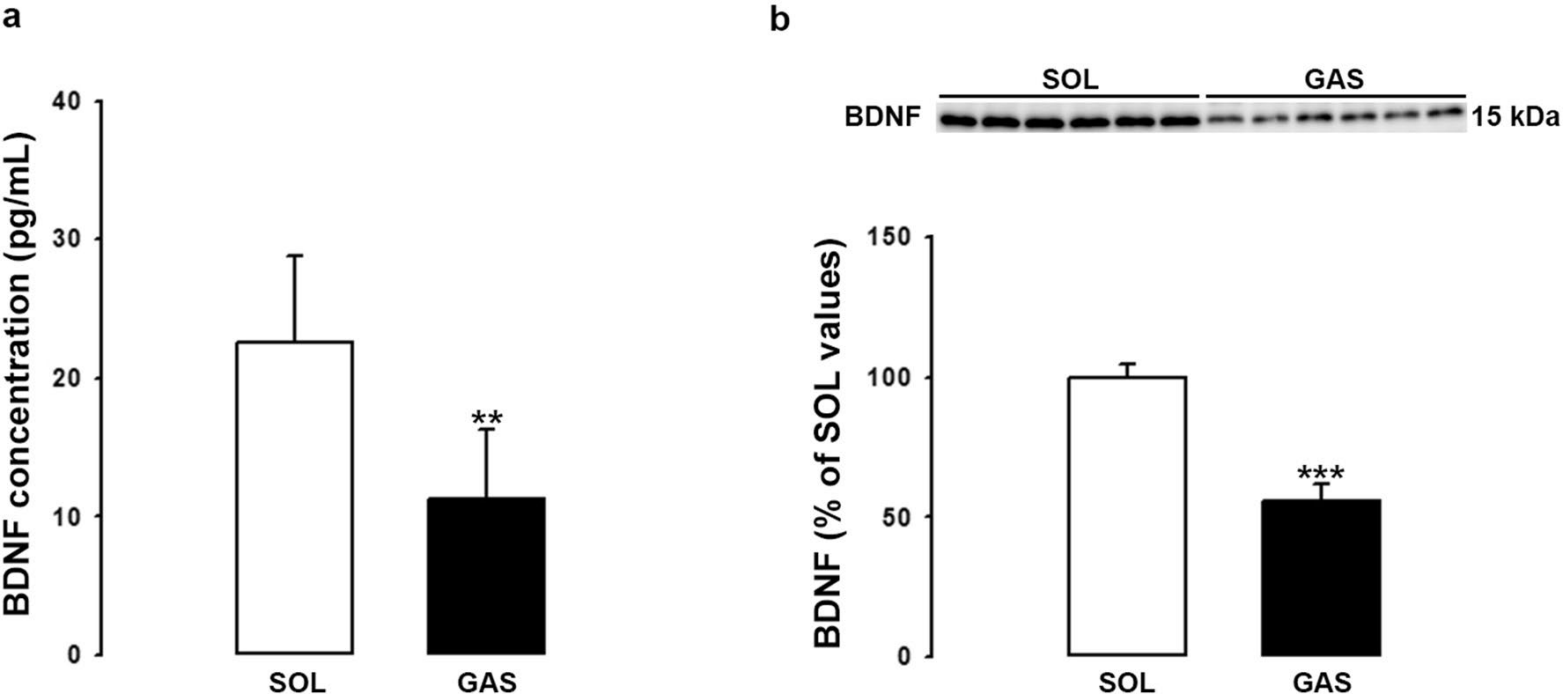
BDNF levels in the soleus (SOL) and gastrocnemius (GAS) muscles. BDNF protein levels in SOL and GAS muscles of control rats (n = 6 per group) were determined by both ELISA (**a**) and Western blotting (**b**) methods. BDNF results (mean ± SD) are expressed in pg/ml (for ELISA) or as percentage of SOL muscle values (for Western blotting). **p < 0.01, ***p < 0.001 versus SOL muscles.

**Figure 5:**
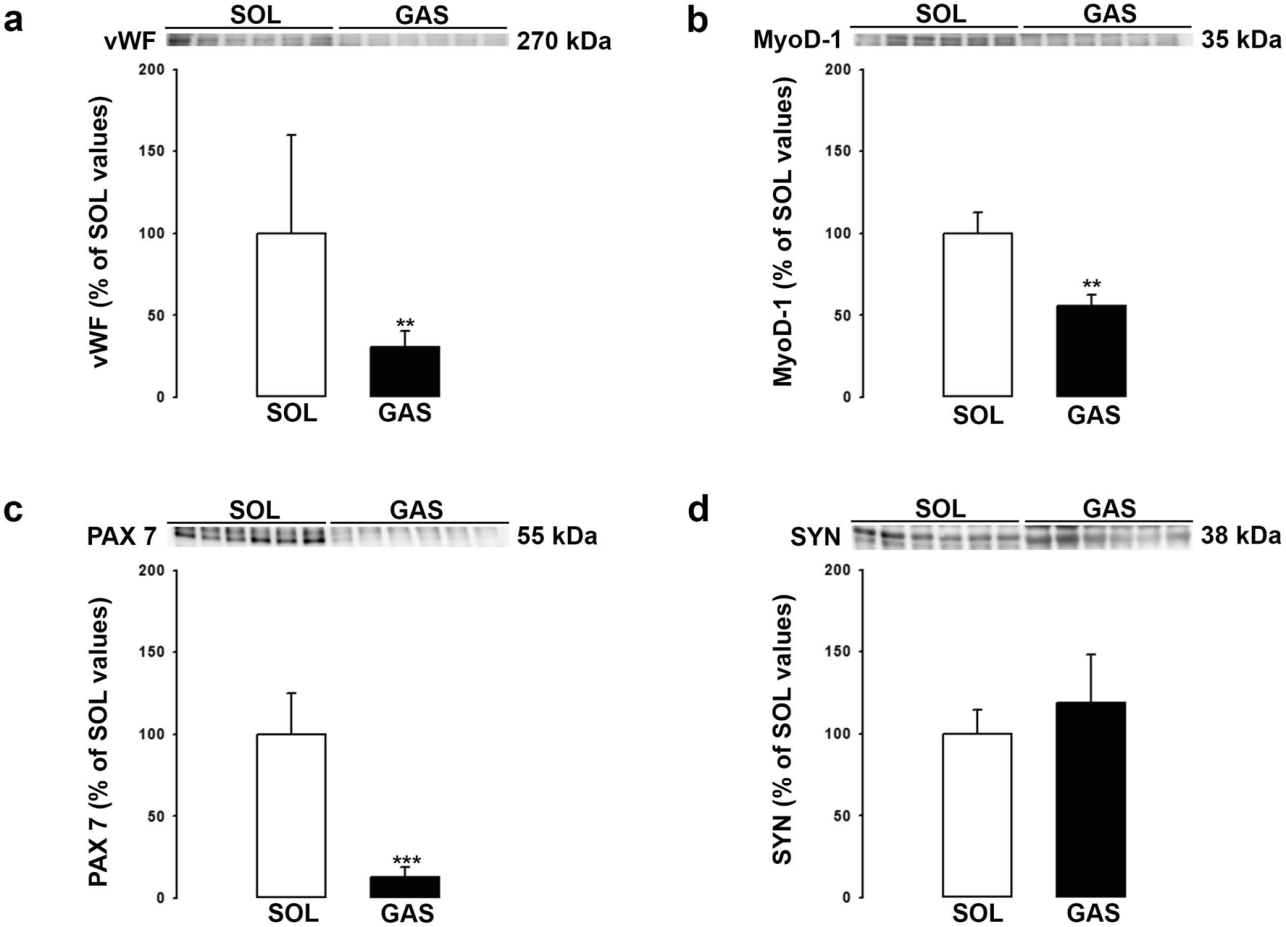
Cellular density of specific markers of endothelial (vWF), satellite (MyoD-1/PAX 7) cells and axonal processes of motor neurons (SYN). Expression of vWF (270 kDa, **a**), MyoD-1 (35 kDa, **b**), PAX 7 (55 kDa, **c**) and SYN (38 kDa, **d**) were determined in SOL and GAS muscles of control rats (n = 6 per group) by Western blotting method. Results are expressed as percentage of SOL muscle values (means ± SD). **p < 0.01, ***p < 0.001 versus SOL muscle. Representative immunoblots of each marker are shown above bar chart.

**Figure 6:**
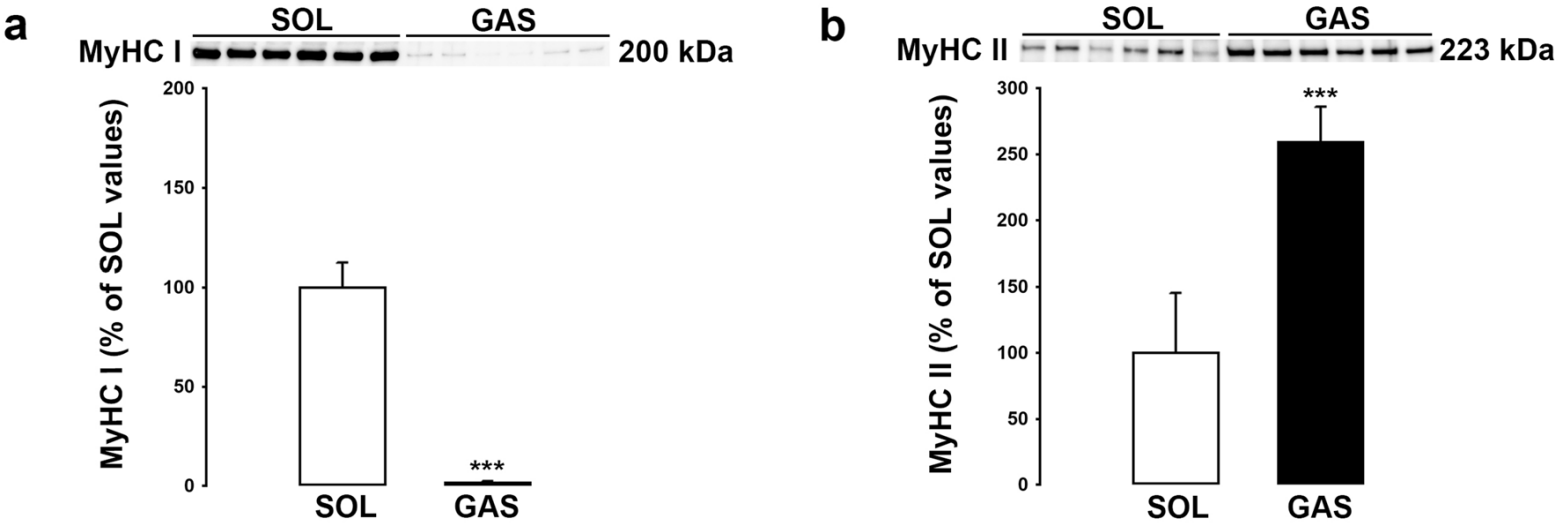
Expressions of slow-type (MyHC I) and fast-type fibers (MyHC II) in soleus (SOL) and gastrocnemius (GAS) muscles. Expression of MyHC I (200 kDa, **a**) and MyHC II (223 kDa, **b**) were determined in SOL and GAS muscles of control rats (n = 6 per group) by Western blotting method. Results are expressed as percentage of SOL muscle values (means ± SD). ***p < 0.001 versus SOL muscle. Representative immunoblots of each marker are shown above bar chart.

The differential expression of BDNF in type II versus type I fibers combined with the strong expression of BDNF by endothelial cells led us to compare BDNF levels in SOL versus GAS muscle. Our hypothesis was that BDNF levels will be higher in SOL than GAS because capillaries and by extension endothelial cells are more abundant in SOL than GAS. BDNF levels were measured using ELISA test and Western blotting method (Fig. 4). As shown in Fig. 4a and b, BDNF levels were significantly higher (X2) in SOL than GAS irrespective of the method used. The cellular density of endothelial cells and of other cells were compared in SOL versus GAS (Fig. 5). The results showed that levels of vWF are threefold higher in SOL than GAS (Fig. 5a). Markers of satellite cells MyoD-1 (Fig. 5b) and PAX 7 (Fig. 5c) were also more abundant in SOL than GAS. In contrast, synaptophysin (SYN) levels (Fig. 5d) did not differ between both muscles. Finally, as expected, marker of type I fibers was higher in SOL than GAS (Fig. 6a) while marker of type II fibers was higher in GAS than SOL (Fig. 6b).

### Activated TrkB levels in muscles

**Figure 7:**
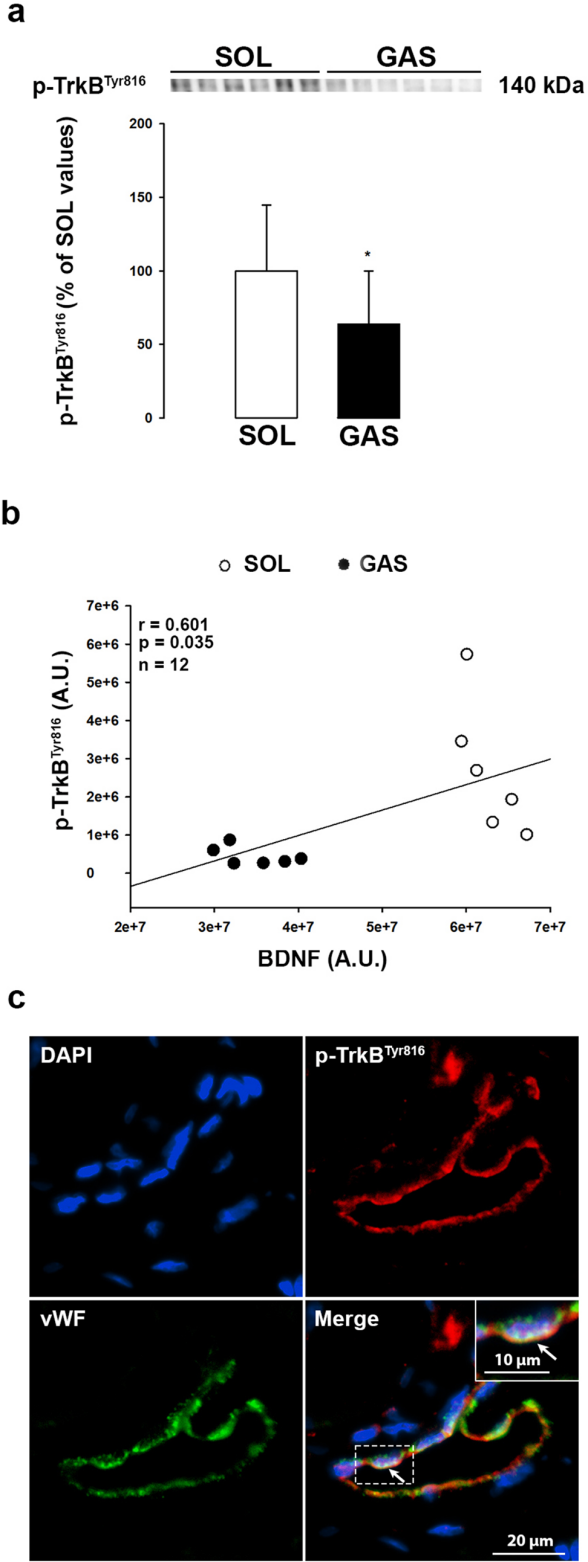
Expression and localisation of p-TrkB^Tyr816^ in SOL and GAS muscles. Correlation with muscular BDNF expression. Expression of p-TrkB^Tyr816^ (140 kDa) was determined in SOL and GAS muscles of control rats (n = 6 per group) by Western blotting method. Results are expressed as percentage of SOL muscle values (means ± SD). *p < 0.05 versus SOL muscle. Representative immunoblot is shown above bar chart (**a**). The correlation (scatter plot) between muscular BDNF and p-TrkB^Tyr816^ was evaluated in SOL and GAS muscles. Values are expressed as arbitrary units (A.U.) and the correlation coefficient (r) as well as the probability (p) are displayed on graph (n = 12) (**b**). Immunostainings of p-TrkB^Tyr816^ and vWF were shown in SOL muscle. DAPI (blue) was used as nuclear marker, red-labelled immunofluorescence represented p-TrkB^Tyr816^ (first line) and green-labelled immunofluorescence, vWF (second line). Similar results were observed in all examinations (*n* = 4), (**c**). The insert shows with higher magnification the colocalization of p-TrkB^Tyr816^ with vWF in presence of DAPI. Scale bar 50 µm (10 µm, for the insert).

As shown in Fig. 7, levels of activated TrkB receptors (p-TrkB^Tyr816^) were higher in SOL than GAS and a correlation was found between BDNF and p-TrkB^Tyr816^ indicating that cellular BDNF secretion was in proportion with BDNF levels. We then explored localization of p-TrkB^Tyr816^ receptors. Immunohistochemical experiments were conducted using antibodies directed against activated TrkB receptors (i.e. FL TrkB receptors activated by BDNF) since antibodies directed against TrkB receptors detect both truncated (incomplete receptor devoid of tyrosine kinase activity) and FL TrkB receptors. As shown in Fig. 7c, these receptors were easily detectable in endothelial cells. By contrast, it was rather difficult to detect their presence in other cell types thus making of endothelial cells a major recipient cell of secreted BDNF.

## Discussion

The present study is the first to provide a comprehensive analysis of BDNF localization in skeletal muscle. The main results are that (i) muscular fibers, satellite cells, motor neurons (axonal processes) and endothelial cells simultaneously express BDNF, yet with a prominent expression by endothelial cells and a more pronounced BDNF expression by type II than type I muscular fibers, (ii) that the muscular expression of activated TrkB receptor is preponderantly attributable to endothelial cells as compared to other cells.

BDNF localization in the skeletal muscle has not been extensively studied. This is surprising as the first detection of BDNF mRNA in skeletal muscle was reported in 1993^6^. In fact, we had to wait for the avaibility of anti-BDNF antibodies to advance research in this field. However, available studies on the localization of BDNF protein led to controversial data. The present study showed that myofibers, satellite cells and axonal processes constitutively expressed BDNF irrespective of the muscle type and that BDNF expression was higher in type II than type I fibers^17,18^. The new finding provided by the present study is the identification of endothelial cells as an important and still unidentified source of BDNF in muscles. Such endothelial BDNF expression in basal conditions likely explains why oxidative muscle exhibits higher BDNF levels than glycolytic muscles despite the higher expression of BDNF by type II fibers^19^. It is noteworthy that BDNF staining in endothelial cells contrasts with a previous study that fails to detect BDNF mRNA in endothelial cells within muscle^8^. In the same way, differential BDNF staining between type I versus type II myofibers contrast with the detection of a higher mRNA BDNF expression in the former. Such discrepancies between mRNA and proteins were previously reported in muscle^18,19^.

TrkB receptors including FL receptors that are equipped with kinase activity and truncated receptors that are devoid of kinase activity are both present in skeletal muscle. The binding of BDNF to FL TrkB receptors and their subsequent phosphorylation has been recently involved in the local effect of muscular BDNF^20–22^. The present study reported the presence of activated TrkB receptors in basal conditions, indicating that there is constitutive secretion of active BDNF by cells. Furthermore, the positive correlation that was identified between muscular BDNF levels and muscular activated TrkB receptors supports the notion that cellular BDNF secretion proportionate with cellular BDNF expression as previously reported for the brain^23^. The localization of activated TrkB receptors on muscular fibers, axonal processes and satellite cells are in line with reports on the role of BDNF on beta oxidation of fatty acids^7^ and the switch from type I to type II fibers^17^, the stability of neuromuscular junction^11,24^ and regulation of satellite cell differentiation^9,25^, respectively. However, the present study showed a prominent endothelial expression of activated TrkB receptors in basal conditions. The cellular source of BDNF involved in the activation of endothelial TrkB receptors might be endothelial cells themselves or neighboring cells including myofibers and satellite cells. The potential impact of activation of endothelial TrkB receptor activation is probably related to the production of nitric oxide (NO), identifying NO as an intermediate between BDNF and muscular function among which muscular metabolism and myogenic differentiation (for review^26–28^).

Endothelial derived BDNF may also account for the elevation of blood BDNF levels in response to physical exercise. Indeed, we previously provide convincing arguments for a positive control of endothelial BDNF by shear stress and subsequent NO overproduction^14–16,29^. Supporting this hypothesis, studies reported lower blood BDNF levels in sedentary than in physically active people^30^ and lower levels in patients at vascular risk, i.e. suffering from endothelial dysfunction^31–33^.

For authors that adhere to the notion that BDNF is a myokine considering that BDNF level elevation during or just after exercise corresponds to BDNF secreted by myofibers, the data presented in our study may lead to reinterpretation of changes in circulating BDNF observed in response to exercise.

In conclusion, our results identified for the first time endothelial cells as a main source of BDNF present in skeleton muscle. Further studies are needed to investigate whether BDNF derived from endothelial cells exerts autocrine, paracrine and/or endocrine role.

## Methods

### Animals

Experiments were carried out on 10 week-old Wistar rats (n = 14) according to the French Department of Agriculture guidelines (License 21-CAE-099) and approved by the local ethic committee (Ethics committee in animal experimentation, Grand campus Dijon, agreement number 105) and conform to ARRIVE guidelines. They conformed to the European convention for protection of vertebrate animals used for experimental and other scientific purposes. The animals were housed five per cage, kept under a 12-h/12-h light/dark cycle and allowed ad libitum access to food and water. Rats were purchased from Janvier (Le Genest Saint Isle, France).

### Collection of muscle samples

Thirty minutes before anesthesia [ketamine (75 mg/kg, Virbac, Carros, France)/xylazine (8 mg/kg Bayer, Leverkusen, Germany), i.p.], rats received a subcutaneous premedication [buprenorphine (0.05 mg/kg, Axience, Pantin, France)]. Anesthetized animals were then transcardially perfused with saline during 5 min to flush out blood from all vasculature. Gastrocnemius (medial head of gastrocnemius, GAS) and Soleus (SOL) muscles were removed from the same paw of rats and quickly dissected on ice-cold glass slide, weighted and frozen at − 80 °C until biochemistry analysis or fixed in 4% paraformaldehyde solution (4% PFA, 9713, VWR) for 48 h and embedded in paraffin until immunofluorescence analysis.

### Biochemistry

Muscles were homogenized in 10 volumes of ice-cold lysis buffer [100 mmol/L Tris–HCl (pH 7.4), 150 mmol/L NaCl, 1 mmol/L EGTA, 1% triton X-100, 1% protease inhibitor cocktail (P8340, Sigma-Aldrich), 1% phosphatase inhibitor cocktail (P1066-8304, Fisher Scientific, only for the study of phosphorylated protein)] with ultraturrax (2 min) and ultrasonic sound (15 s). After homogenization, the protein concentration was measured using the Lowry method.

### Elisa test

BDNF protein levels were determined with a commercial ELISA kit (BEK-2211-2P, Biosensis). According to the manufacturer, BDNF antibodies do not cross react with nerve growth factor (NGF), neurotrophin-3 (NT-3), NT-4/5. The limit of sensitivity was fixed at 2 pg/ml. The measurements were performed according to the manufacturer’s instructions. Supernatants of muscles homogenates were directly dropped on the plate without dilution. All assays were performed in duplicate.

### Western blot

Equal amounts of protein were loaded on sodium dodecyl sulfate–polyacrylamide (SDS-PAGE) TGX Stain-Free FastCast gel electrophoresis (TGX Stain-Free FastCast Acrylamide Kit, 7.5%, 161-0181 and 12%, 161-0185, Bio-Rad). According to BioRad protocol, after electrophoresis (30 min, 300 V), gels containing proteins were activated to UV light with Chemidoc imaging systems (12003153, Bio-Rad) using “Stain free gel activation (45 s)” program. Then, gels were electrophoretically transferred to polyvinylidene difluoride (PVDF) membranes at 1.3 A constant, up to 25 V using Turbo Transblot technology (1704150, Biorad). After blocking non-specific binding sites for 45 min, with a 5% solution of non-fat dry milk or 7.5% BSA in Tris Buffered Saline TBS (20 mM Tris/HCl, 137 mM NaCl, pH 7.4) containing 0.1% Tween 20 (TBST), membranes were probed with an anti-BDNF (1/3000, rabbit monoclonal, ab108319, Abcam), anti-vWF (1/2000, rabbit monoclonal, F3520, Sigma-Aldrich), anti-MyoD-1 (1/500, mouse monoclonal, ab16148, Abcam), anti-PAX 7 (1/2000, mouse monoclonal, ab218472, Abcam), anti-SYN (1/3000, rabbit polyclonal, RB-1461-P1, Interchim), anti-p-TrkB^Tyr816^ (1/1000, rabbit polyclonal, ABN1381, Millipore), anti-p-eNOS^Ser1177^ (1/1000, mouse monoclonal, 612,383, BD Biosciences) antibody for 1h30 at room temperature. Membranes were then incubated at room temperature (1 h) with horseradish peroxidase antibody [111-035-144 (anti-rabbit) or 115-035-166 (anti-mouse), 1/10000 to 1/50000 according to the protein, Jackson ImmunoResearch]. After antibody incubation, membranes were placed in Chemidoc imaging systems and a stain free image of the blot was captured to control the total protein loading and normalize data. Protein-antibody complexes were visualized using the enhanced chemiluminescence western blotting detection system (ECL 2, 1151-7371, Fisher Scientific) and Chemidoc imaging systems. Band densities were finally analyzed with ImageLab software (Bio-Rad) and standardized on total protein. The appropriate amounts of total proteins to be analyzed were previously determined from concentration (increasing amounts of proteins)/response (optical density of the band) curves from two rats both belonging to a particular group (on the same gel). All gels were run in triplicate.

### Histology

Experiments were carried out in CellimaP corefacility, INSERM LNC-UMR1231, Dijon, France. Briefly, muscles collected in anaesthetized rats were fixed during 48 h in 4% PFA solution (9713, VWR) and dehydrated in alcohol baths according to this order: Ethanol 70° (1 bath of 30 min), Ethanol 95° (3 baths of 60 min) and finally, Ethanol 100° (3 baths of 60 min). Samples were then incubated in Methylcyclohexane (3 baths of 60 min) and embedded in paraffine (3 baths of 2 h). Every step was realized in a dehydration automaton (ASP300, Leica). Muscles sections (5-µm) were cut with a microtome (RM2245, Leica), collected on SuperFrost Plus slides (Thermo Fisher) and dried overnight at 45 °C.

### Immunofluorescence

Slices were deparaffinized and rehydrated in different successive baths of xylene and ethanol, then washed in TBS. Heat-induced epitope retrieval was then realized in Sodium Citrate Buffer (Trisodium citrate, 10 mmol.L-1, 0.1% Tween 20, pH 6.0) or Tris–EDTA Buffer (Tris 10 mmol.L-1, EDTA 1 mmol.L-1, 0.1% Tween 20, pH 9.0) in water bath during 20 min at 95 °C. After blockade of non-specific binding sites with TBST solution containing 3% of goat serum (GS) for 30 min at room temperature (RT), slides were first incubated overnight at 4 °C with TBST solution containing 1% of GS and specific antibody : anti-BDNF (1/200, rabbit, monoclonal, ab108319, Abcam), anti-Slow Skeletal Myosin Heavy Chain (1/200, mouse, monoclonal, ab11083, Abcam), anti-Fast Skeletal Myosin Heavy Chain (1/300, mouse, monoclonal, ab51263, Abcam), anti-Pax7 (1/50, mouse, monoclonal ab199010, Abcam), anti-Myo-D1 (1/50, mouse, monoclonal, ab16148, Abcam), anti-synaptophysin (1/200, mouse, monoclonal, MA1-213, Invitrogen, Carlsbad, CA, USA), anti-p-TrkB^Tyr816^ (1/200, rabbit, polyclonal, ABN1381, Millipore) or anti-vWF (1/200, mouse, monoclonal, US Biological, 142,828). Every immunofluorescence was realized with a negative control, i.e. slice without primary antibody, to visualize eventual nonspecific background staining and tissue autofluorescence. Muscle slices were then exposed to a couple of fluorescent secondary antibodies: Alexa Fluor 488 and 568 [A11029 (488 anti-mouse) and A11036 (568 anti-rabbit), or A11034 (488 anti-rabbit) and A11031 (568 anti-mouse), Molecular Probes, Invitrogen], 1/1000 in TBST with 1% of GS, for 60 min at RT. Finally, slides were mounted with DAPI (a nuclear marker)-containing mounting medium (Fluoro-gel with DAPI, FP-DT094A, Interchim, Montluçon, France). For vWF + BDNF and Pax7 + BDNF co-staining, slides were treated with an autofluorescence quenching kit (Vector® TrueVIEW®, SP-8500, Vector Labs, Burlingame, CA, USA) prior to mounting with the DAPI-containing mounting medium of the kit. Slides were analyzed by using an epifluorescent microscope (Axioscop 40FL, Carl Zeiss, Oberkochen, Germany) and ProView v4.1 (Optika, Ponteranica, Italy).

Concerning immunostaining directed against BDNF, it is also important to notice that regarding some unspecific bands obtained in WB assessment, the fluorescent signal may represent some unspecific labelling. However, data obtained in mice with tie-2 conditional deletion of BDNF showing that BDNF staining totally disappears in the endothelium of these animals (see Fig. 4 of supplemental data files) comfort us in the reliability of the antibody used in our study.

### Data and statistical analysis

SigmaPlot 14.0 was used for statistical analysis and all graphs. Data were expressed as means ± standard deviations (SD) for protein expression. Differences between two groups were assessed using parametric t-test or non-parametric Mann–Whitney test, depending on the normality and equal variance tests. T-test for Pearson’s correlations were used to measure the strength of the relationship between paired data. A value of p < 0.05 was considered statistically significant.

## Supplementary Information

Supplementary Information 1.

Supplementary Information 2.

Supplementary Information 3.

Supplementary Information 4.

## Data Availability

The data support the findings of this study are available from the corresponding author upon reasonable request.
