# Supplementary figures and images for: Endothelial cells are an important source of BDNF in rat skeletal muscle
Source: Sci Rep. 2022 Jan 10;12:311. doi: 10.1038/s41598-021-03740-8 (PMC8748777; doi:10.1038/s41598-021-03740-8)

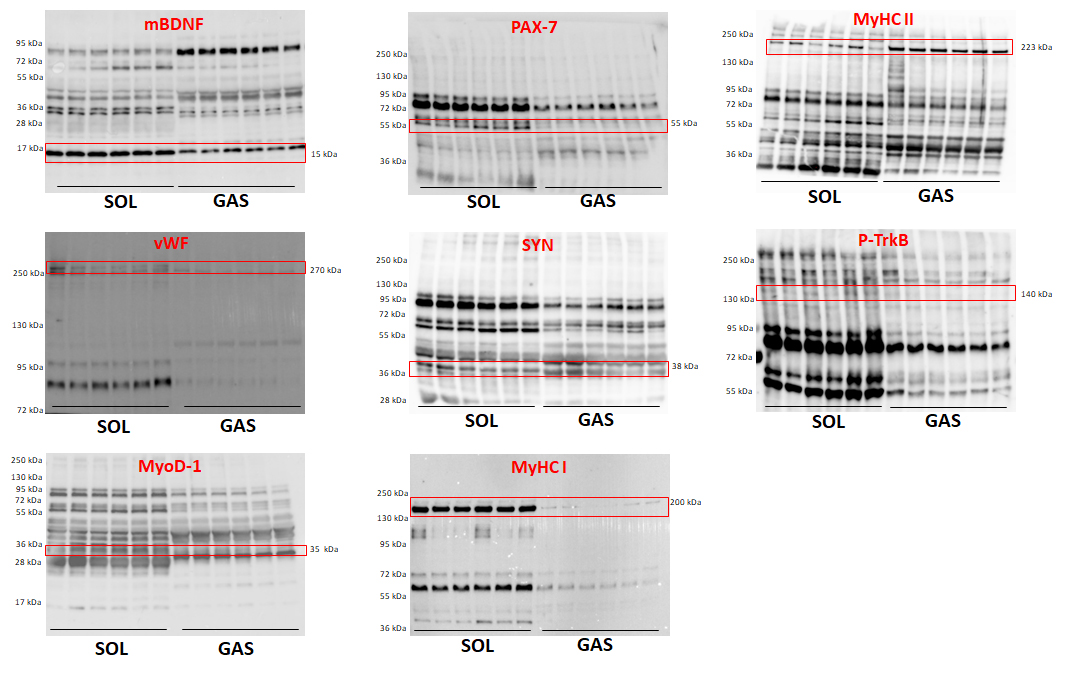

Supplement: Supplementary file 1 — Supplementary Information 1. [file 41598_2021_3740_MOESM1_ESM.jpg]

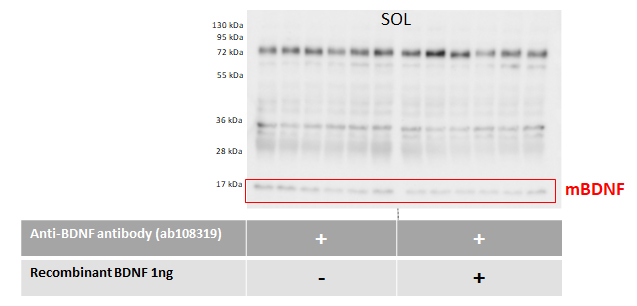

Supplement: Supplementary file 2 — Supplementary Information 2. [file 41598_2021_3740_MOESM2_ESM.jpg]

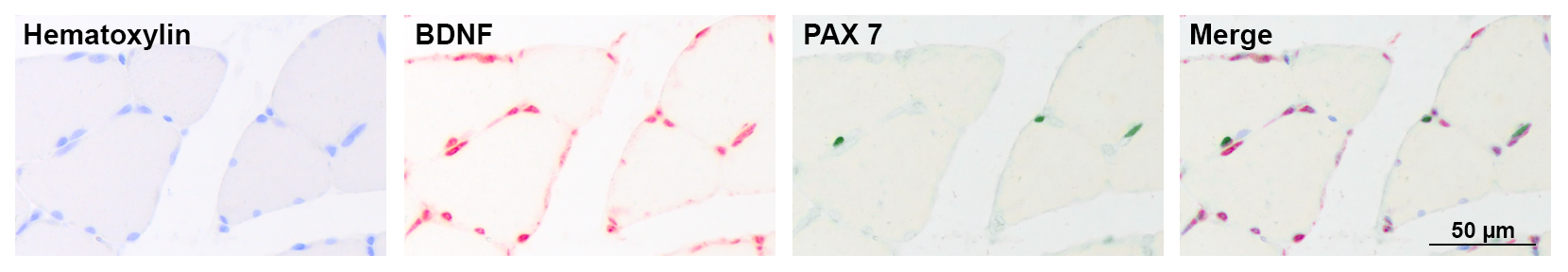

Supplement: Supplementary file 3 — Supplementary Information 3. [file 41598_2021_3740_MOESM3_ESM.jpg]

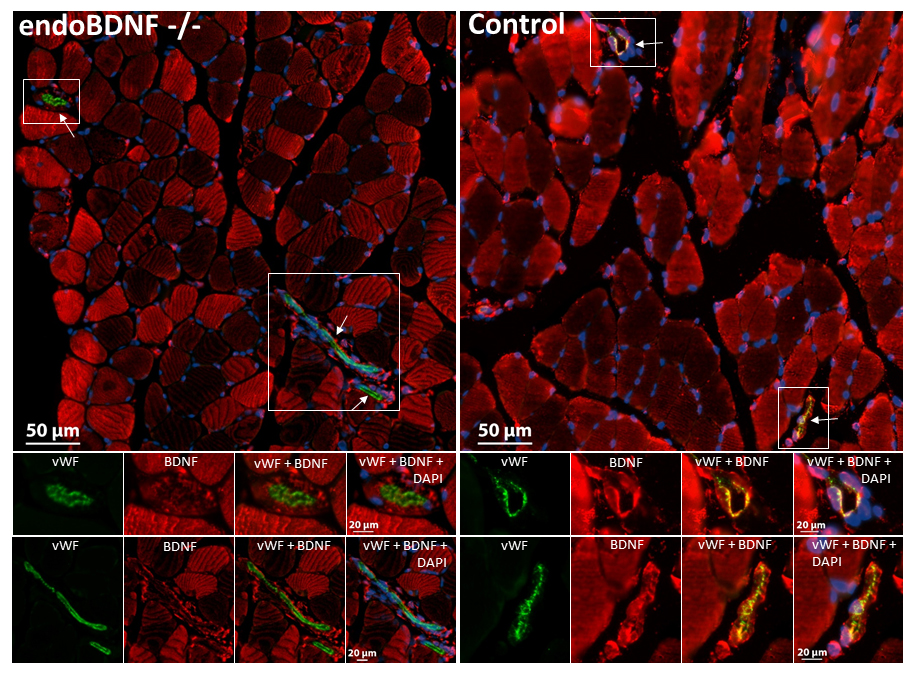

Supplement: Supplementary file 4 — Supplementary Information 4. [file 41598_2021_3740_MOESM4_ESM.jpg]
